# Fabrication and Characterization of Isotropic and Anisotropic Magnetorheological Elastomers, Based on Silicone Rubber and Carbonyl Iron Microparticles

**DOI:** 10.3390/polym10121343

**Published:** 2018-12-05

**Authors:** Jesús G. Puente-Córdova, M. Edgar Reyes-Melo, Luis M. Palacios-Pineda, Imperio A. Martínez-Perales, Oscar Martínez-Romero, Alex Elías-Zúñiga

**Affiliations:** 1 Faculta de Ingeniería Mecánica y Eléctrica, Universidad Autónoma de Nuevo León, Av. Universidad s/n, Ciudad Universitaria, 66451 San Nicolás de los Garza, Mexico; jesus.puentecr@uanl.edu.mx (J.G.P.-C.); edgar.reyesml@uanl.edu.mx (M.E.R.-M.); 2 División de Estudios de Posgrado e Investigación, Tecnológico Nacional de México/Instituto Tecnológico de Pachuca, Carr. México-Pachuca Km 87.5, 42080 Pachuca, Mexico; palacios@itpachuca.edu.mx; 3 Escuela de Ingeniería y Ciencias, Tecnologico de Monterrey, Ave. Eugenio Garza Sada 2501, 64849 Monterrey, Mexico; anel.perales@itesm.mx (I.A.M.-P.); oscar.martinez@itesm.mx (O.M.-R.)

**Keywords:** magnetorheological elastomer, carbonyl iron microparticles, Cole-Cole diagram, fractional Zener model

## Abstract

This article focuses on studying the rheological behavior of isotropic and anisotropic magnetorheological elastomers (MREs), made of carbonyl iron microparticles dispersed into a silicone–rubber matrix by considering 20 and 30 wt % of microparticles. Sample sets were prepared for each composition, with and without the application of an external magnetic field. Experimental measurements of the material rheology behavior were carried out by a shear oscillatory rheometer at constant temperature, to determine both the shear storage modulus (G′) and shear loss modulus (G′′) for all characterized samples. Then, experimental data collected from the isotropic and the anisotropic material samples were used to plot the Cole-Cole diagrams to quantify the interfacial adhesion between carbonyl iron microparticles and the silicone-rubber matrix. Furthermore, the Fractional Zener Model (FZM) with two spring-pots in series is used for quantitative analysis of collected experimental data.

## 1. Introduction

Magnetorheological elastomers (MREs) are materials that can exhibit variable stiffness and damping properties if subjected to the action of an external magnetic field. These are used in several engineering applications to widen the bandwidth value in tunable vibration absorbers [1,2,3], in machine isolators [4,5], and to sense mechanical and magnetic signals [6], to name a few.

It is also well-known that the mechanical performance of these MRE materials could be improved by the incorporation of carbonyl iron microparticles as a material reinforcement [7,8]. The MREs studied in this paper are composed of silicone rubber, reinforced with carbonyl iron microparticles that could be aligned or not, i.e., isotropic MRE samples do not have magnetic microparticle alignment, while anisotropic MREs could have magnetic microparticles aligned parallel to the magnetic field lines that can enhance their physical properties. For instance, Sohoni [9] manufactured anisotropic MREs and found that the alignment of the reinforced particles tends to improve the mechanical properties of the composite material, in comparison with those that have an isotropic distribution of the magnetic particles. Later, these findings were confirmed by Li in [10]. In fact, the chain-like structures induced in anisotropic MRE materials enhance their dynamic stiffness and damping properties [11]. However, Moucka et al. [12] used dielectric spectroscopy to study the extent of magnetic filler within the polymer matrix, by applying a static magnetic field during the curing process. They observed that the chain-like clusters of MREs were linked to dielectric relaxations because of the charge transport via the variable range hopping mechanism, something that was not previously observed in isotropic distribution of the magnetic particles. They concluded that increasing filler concentration shortens the mean relaxation time but enhances particle distribution into the polymer matrix material.

On the other hand, it is also well-known that when magnetic particles are added into soft or hard elastomeric matrices, the resulting MRE composite materials exhibit a significant variation of their mechanical properties upon the application of a magnetic field. In fact, anisotropic MREs with a soft matrix show large magneto-rheological effects when compared to those produced with hard elastomeric matrices.

To model the anisotropic viscoelastic behavior exhibited by MREs, several mathematical models have been proposed, such as the one introduced by Rudykh [13]. Rudykh’s proposed model is able to predict the increase in material stiffness induced by an applied magnetostatic excitation to anisotropic MREs, by using explicit expressions for the macroscopic response. By using the Zener model, Metzler and Nonnenmacher [14] found a relationship between the fractional Fokker–Planck equation, and the anomalous relaxation dynamics of a class of viscoelastic materials that exhibit scale-free memory. Bartkowska and co-workers [15] applied the fractional Zener model with two spring-pots, to describe the relaxation time spectrum of ferroelectric ceramic material. They derived an enhanced model to overcome the shortcomings of the fractional Zener model with one spring-pot. Sebald [16] investigated the viscoelastic behavior and the pseudo-Villari effect exhibited by MREs materials, and used dynamic viscoelastic expressions to predict recorded stress–strain experimental data. Poojary and co-workers [17] considered an integer and fractional order derivative approaches to model the viscoelastic response behavior of MREs by using the two-Maxwell and the fractional Maxwell models, respectively. They were able to represent the field induced between the range of 0 T to 0.27 T, as well as the dynamic compression characteristic for the frequency range of 8–24 Hz.

Motivated by these findings, the aim of this paper focuses on using the Fractional Zener Model (FZM) to predict the shear storage modulus (G’) and the material shear loss modulus (G’’) of a magnetorheological silicone–rubber matrix reinforced with carbonyl iron microparticles. It is proved that the FZM material model captures the influence that the particle alignment has on the reinforced material, since theoretical predictions follow experimental data well.

## 2. A Brief Introduction of Fractional Calculus in Polymer Rheology

Fractional calculus is a mathematical tool that deals with the derivatives and integrals of arbitrary order [18]. In the pioneering work published by Gemant in 1936 [19], the behavior of elasto-viscous bodies was studied by using a half differential equation relating stress, strain, and time. Later, Padovan and Guo [20] investigated the impulse-transient-steady-mixed solution properties of a fractionalized Maxwell–Kelvin Voigt-type viscoelastic response. Unlike the integer case, which displays a hyper-linear relationship between the force and velocity, acceleration, and the strain rate, they found that the fractional representation is nonlinear with respect to these quantities. Additionally, Schiessel et al. [21] replaced the linear elastic and viscous elements with fractional ones, while keeping the number of the parameters involved relatively low. Based on the evidence provided by the aforementioned articles and references cited therein, a fractional calculus approach is needed to properly model the viscoelastic behavior of polymers [22,23,24].

To achieve a fractional order derivative in the material constitutive equations, dashpots in rheological models can be replaced by a fractional order using a spring-pot element. The spring-pot intimately combines the solid behavior (Hookean spring) with liquid behavior (Newtonian dashpot) by a differential operator of the fractional order in the form

(1) $$\sigma =G\tau^{a}D_{t}^{a}\gamma ,$$

where σ, γ, and *G* are the shear stress, strain, and modulus, respectively; τ=η/G represents the relaxation time, which could be associated with the time required by the movement of the segments of chains for a complete reorganization and reorientation to a new structural equilibrium state; η is the material viscosity; and $D_{t}^{a}\gamma$ is the derivative of the *a*th order (0≤a≤1) of the deformation with respect to time. According to Riemman–Liouville, the fractional derivative (of a-th order) in Equation (1) is defined as

(2) $$D_{t}^{a}\gamma =\frac{1}{\Gamma \left(1-a\right)}\frac{d}{dt}\int_{0}^{t}{\left(t-y\right)}^{-a}\gamma \left(y\right)dy,$$

and the fractional integral, defined between 0 and *t*, can be expressed by

(3) $$D_{t}^{-a}=\int_{0}^{t}\frac{{\left(t-y\right)}^{a-1}}{\Gamma \left(a\right)}\gamma \left(y\right)dy,$$

where Γ(x) is the Gamma function, which follows the definition

(4) $$\Gamma \left(x\right)=\int_{0}^{\infty }\left(e^{-u}u^{x-1}\right)du\;\mathrm{with}\;x>0$$

**Figure 1:**
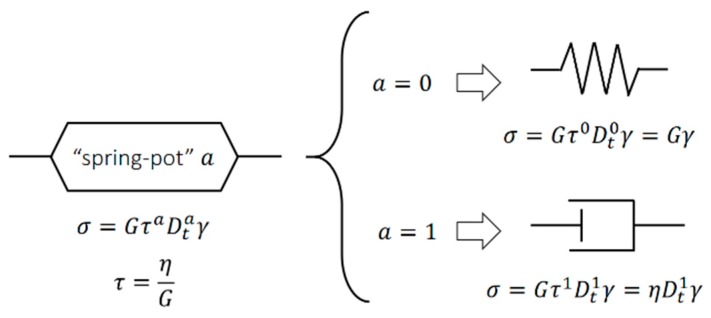
The spring-pot element.

When a=0 in Equation (1), one obtains the Hooke’s law or spring behavior, but if a=1 then, one obtains Newton’s law or dashpot material behavior, as shown in Figure 1.

From the physical point of view, the fractional order of Equation (2) reflects the rate at which a portion of the energy is lost in the viscoelastic system. Similarly, the fractional order of Equation (3) is an indication of the remaining energy of a signal that is passing through a viscoelastic material. The relationship between the lost and the stored energies are proportional to the shear loss modulus $\left(G^{\prime \prime }\right)$ and to the shear storage modulus $\left(G^{\prime }\right)$, respectively, and both can be represented by the complex shear modulus, $G^{*}=G^{\prime }+iG^{\prime \prime }$. The experimental measurements of the real and imaginary parts of $G^{*}$ for polymer samples could be carried out by oscillatory shear experiments using the parallel-plate geometry [25,26].

Since a single Zener spring-pot model is not able to predict the asymmetrical shape of the experimental curves of the real and imaginary parts of $G^{*}$ that describe the rheological polymer behavior [27], the Fractional Zener Model (FZM), which has two spring-pots, must be used to predict the behavior exhibited by the $G^{\prime }$ and $G^{\prime \prime }$ curves [15,16,22,24]. This FZM will be briefly review in the next section.

## 3. Modeling of the Complex Shear Modulus $G^{*}$

**Figure 2:**
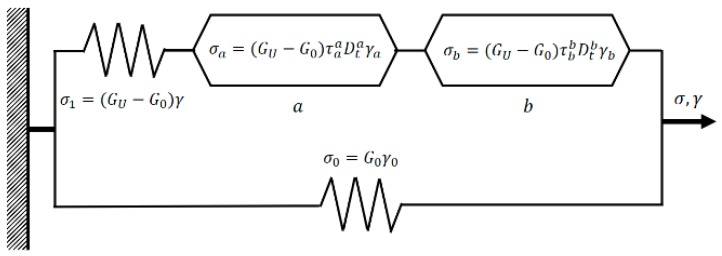
The Fractional Zener Model (FZM), composed of two springs and two spring-pots (*a* and *b*).

The classical Zener model can be modified by replacing the dashpot by two spring-pots in series a and b. Figure 2 shows the three components that describes the FZM. First, the spring-pot, a, characterizes short times ($\tau_{a}$) associated with viscoelastic behavior in the region of high frequencies. The spring-pot, b, characterizes long times ($\tau_{b}$) associated with viscoelastic behavior in the region of low frequencies, and the two spring elements represent the elastic polymer behavior.

In Figure 2, $\tau_{a}$ and $\tau_{b}$ are the relaxation times of the spring-pots a and b, $G_{0}$ is the relaxed modulus corresponding to values of $G^{\prime }$ at low frequencies, $G_{U}$ is the unrelaxed modulus corresponding to the values of $G^{\prime }$ at high frequencies. Therefore, and from the constitutive equations of springs and spring-pot elements exhibited in Figure 2, the differential equation of the non-integer order for the FZM can be written as

(5) $$\left(G_{U}-G_{0}\right)\gamma =\left(\sigma -G_{0}\gamma \right)+\tau_{b}^{-b}D_{t}^{-b}\left(\sigma -G_{0}\gamma \right)+\tau_{a}^{-a}D_{t}^{-a}\left(\sigma -G_{0}\gamma \right)$$

From Equation (5), and by considering that the polymer sample is under a mechanical stimulus that follows a sinusoidal form, the $G^{*}$ can be calculated as a function of the angular frequency ω at a constant temperature, by applying the Fourier transform to Equation (5) [22,28,29]. This yields

(6) $$G^{*}\left(i\omega \right)=G^{\prime }+iG^{\prime \prime }=\frac{G_{U}+G_{0}\left[{\left(i\omega \tau_{a}\right)}^{-a}+{\left(i\omega \tau_{b}\right)}^{-b}\right]}{1+{\left(i\omega \tau_{a}\right)}^{-a}+{\left(i\omega \tau_{b}\right)}^{-b}}$$

and by separating the real and imaginary terms, the expressions for $G^{\prime }$ and $G^{\prime \prime }$ are given as

(7) $$\begin{array}{cc} G^{\prime } & =G_{0}+\frac{\left(G_{U}-G_{0}\right)\left(1+A_{1}\right)}{{\left(1+A_{1}\right)}^{2}+A_{2}^{2}},\\ G^{\prime \prime } & =\frac{\left(G_{0}-G_{U}\right)A_{2}}{{\left(1+A_{1}\right)}^{2}+A_{2}^{2}}, \end{array}$$

where

(8) $$\begin{array}{l} A_{1}={\left[\omega \tau_{b}\right]}^{-b}cos\left(b\frac{\pi }{2}\right)+{\left[\omega \tau_{a}\right]}^{-a}cos\left(a\frac{\pi }{2}\right),\\ A_{2}={\left[\omega \tau_{b}\right]}^{-b}sin\left(b\frac{\pi }{2}\right)+{\left[\omega \tau_{a}\right]}^{-a}sin\left(a\frac{\pi }{2}\right). \end{array}$$

**Figure 3:**
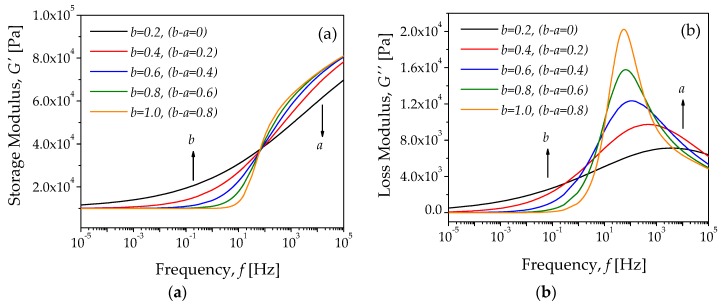
Theoretical diagrams computed from the FZM for several values of parameter b, with parameter values of a = 0.2, $G_{0}$ = 1 × 10^4^ Pa, $G_{U}$ = 1 × 10^5^ Pa, $\tau_{a}$ = 0.001 s, and $\tau_{b}$ = 0.002 s. (**a**) storage modulus $G_{\prime }$ and (**b**) loss modulus $G^{\prime \prime }$.

From Equations (7) and (8), theoretical isothermal diagrams were constructed for $G^{\prime }\left(f\right)$ and for $G^{\prime \prime }\left(f\right)$, with the purpose of identifying the way in which the fractional parameters a and b defined the shape of the computed curves. Here *f* is the frequency expressed in Hertz. To compute these theoretical curves, one could vary the fractional order values of a and b that constitute the FZM. The remaining parameter values were selected in a heuristic way. It is important to note that both fractional parameters a and b can only take values between 0 and 1, and the minimum and maximum values correspond, respectively, to the spring element and the dashpot element. Figure 3a shows the storage modulus $G^{\prime }\left(f\right)$, while Figure 3b exhibits the corresponding loss modulus $G^{\prime \prime }\left(f\right)$.

**Figure 4:**
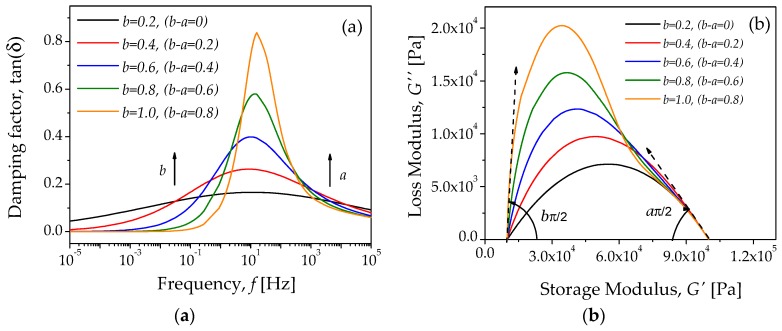
Theoretical curves: (**a**) $\tan\delta =\frac{G^{\prime \prime }}{G^{\prime }}$ and (**b**) the Cole–Cole diagram computed from Equation (7) for *a* = 0.2, and several parameter values of *b*. The parameter values of $G_{0}$, $G_{U}$, $\tau_{a}$, and $\tau_{b}$ are 1 × 10^4^ Pa, 1 × 10^5^ Pa, 0.001 s, and 0.002 s, respectively.

Two additional diagrams can be plotted by using Equation (7). The first corresponds to the frequency dependence of $\tan\delta =G^{\prime \prime }/G^{\prime }$, as illustrated in Figure 4a; the second one represents the Cole–Cole diagram, as shown in Figure 4b. The peaks observed in the tanδ curves correspond to the damping terms of the FZM, which are related to the mechanical relaxation peaks, while Figure 4b shows how the slope of the Cole–Cole diagram can be used to predict the fractional parameter values of a and b. The theoretical curves obtained for both $G^{\prime }$ and $G^{\prime \prime }$ could be compared with experimental data to describe the main relaxation process of amorphous polymeric materials, as discussed in [30,31,32]. Notice that $G^{\prime }$ is a curve that depends on the frequency. Moreover, the shape of curves for tanδ and the Cole–Cole diagrams are also similar to experimental curves reported in the literature [32].

The theoretical curves exhibited in Figure 3 and Figure 4 were plotted by keeping constant the value of a, and by varying the parameter value of b with $\tau_{a}<\tau_{b}$. Notice from Figure 3 and Figure 4 that the curves become more asymmetric as the difference between the fractional parameters a and b increases.

## 4. Experimental Results

### 4.1. Material Synthesis and Characterization

**Figure 5:**
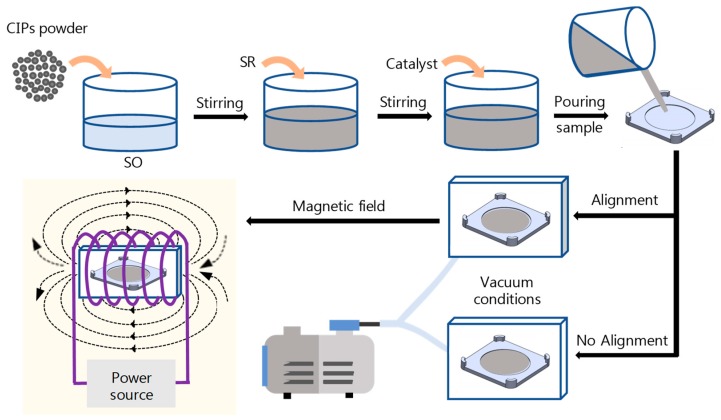
Infographic showing the steps followed to obtain the magnetic particle-reinforced composite material samples.

The materials used to manufacture the MREs were silicone oil (SO) with a viscosity value of 0.25 Pa·s, silicone rubber RTV 3325 (SR) with a viscosity value of 40 Pa·s, and tin catalyst (TC), all purchased from ChemSil (México City, México). The spherical carbonyl iron particles, with an average size of 2.5 µm, were purchased from Sigma-Aldrich (Monterrey, México). The composite materials were prepared by following the procedure steps described in [8]. Figure 5 illustrates the procedure infographics. Two different concentrations of magnetic particles were considered—20 and 30 wt %—since we have found that at about these concentration values, the composite material exhibits its best mechanical performance [8]. First, the magnetic particles were immersed in SO and mixed for a couple of minutes, then the SR was added, considering the proportion of SR:SO:TC as 20:1:1. All the constituents were mixed at room temperature during 5 min. The high viscosity of the solution requires a high speed of the mixing process, to avoid sedimentation of the added magnetic particles. The homogeneous mixture was placed into a mold, and the curing process was carried out at room temperature for 12 h, and under vacuum conditions to avoid porosity. During the curing process, only the anisotropic reinforced samples were exposed to a magnetic flux density of 7 mT. It is important to mention that after 20 min of the curing process, the mixture practically reached a semi-solid state, which hindered magnetic particle sedimentation [33].

**Figure 6:**
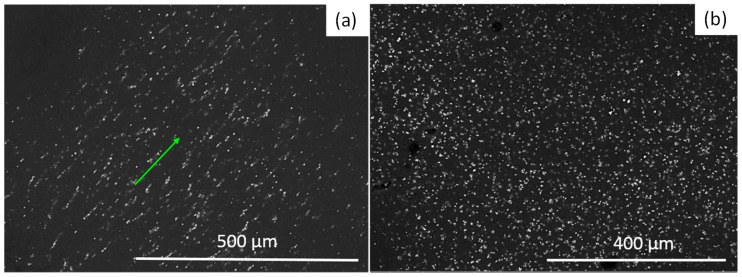
Scanning electron microscopy (SEM) images of the developed magnetorheological elastomer (MRE) material samples: (**a**) anisotropic sample and (**b**) isotropic material sample.

To evaluate the homogeneity of the developed composite material and its particle alignment, the samples were analyzed by scanning electron microscopy (SEM, Quanta 250-FEG FEI, Thermo Fisher Scientific, Waltham, MA, USA). Figure 6a shows the particle alignment exhibited by the anisotropic samples, while Figure 6b illustrates an isotropic particle distribution of the composite material. Also notice from Figure 6 that the isotropic sample exhibits a better homogeneity distribution than the anisotropic one. This can be explained from the particle arrangement induced by the low magnetic field applied during the curing process.

**Figure 7:**
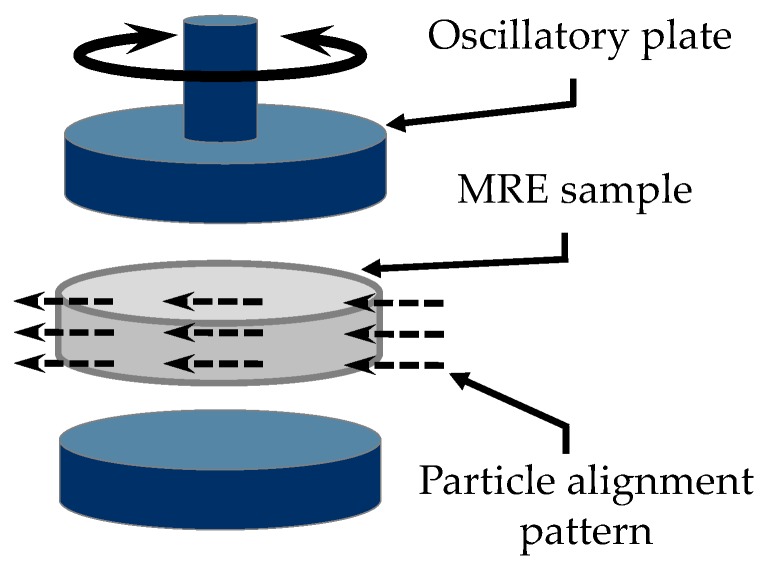
Perpendicular particle alignment pattern for the anisotropic MRE sample with respect to the oscillatory plate rotation axis.

The shear storage ($G^{\prime }$) and the shear loss ($G^{\prime \prime }$) moduli were measured by using an Anton Paar rheometer, model MCR301 (Anton Paar GmbH, Graz, Austria). A parallel-plate rotor was installed in the rheometer, and all experimental tests were performed at room temperature (25 °C) using 10 mm diameter cylindrical specimens with 1 mm thickness. Notice from Figure 7 that the particle alignment pattern of the anisotropic samples is perpendicular to the axis of the rheometer moving upper plate. Each material sample was subjected to an angular shear-mode harmonic motion, and a rotating moment was acting on the moving upper part of the measuring unit in the regime of dynamic oscillations, subjected to a frequency range of 0.01 to 100 Hz, in order to induce a maximum shear strain value of 1%. Because of the sample sizes and material density, the samples’ natural frequencies were far from resonance conditions. Also, a normal constant force of 1.5 N was applied on the sample surface, to avoid possible slippage wall effects.

### 4.2. Complex Shear Modulus $G^{*}$ Experimental Results

**Figure 8:**
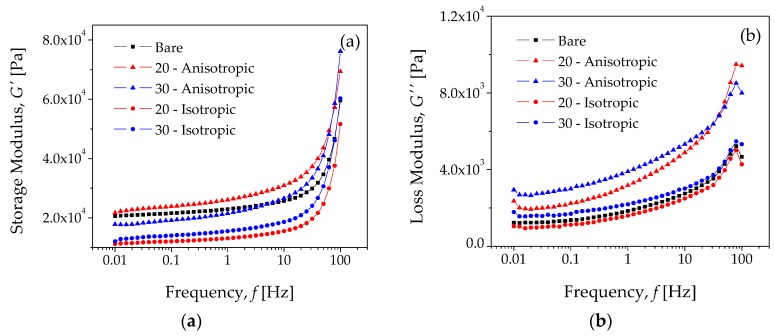
Experimental curves for the bare and composite MRE samples: (**a**) the storage modulus, $G^{\prime }$, and (**b**) the loss modulus, $G^{\prime \prime }$.

The experimental curves of $G^{\prime }$ and $G^{\prime \prime }$ for the bare and composite MRE samples are shown in Figure 8. In all cases, the $G^{\prime }$ showed a slightly increasing trend when frequency was in the lower range. However, when the frequency was above a critical value (~30 Hz), the shear storage modulus starts to increase abruptly, and thus, the sample experienced a shear-stiffening effect [30]. Over the analyzed frequency range (from 0.1 to 100 Hz), in the bare sample, the $G^{\prime }$ value goes from 20.6 kPa to 59.6 kPa, which represents an increase of 189.5%. Also notice from Figure 8b that $G^{\prime \prime }$ increases to the frequency value of 79 Hz, and then its value decreases. At the maximum value of $G^{\prime \prime }$, the mechanical peak relaxation occurs, which corresponds to the maximum material sample energy dissipation.

**Figure 9:**
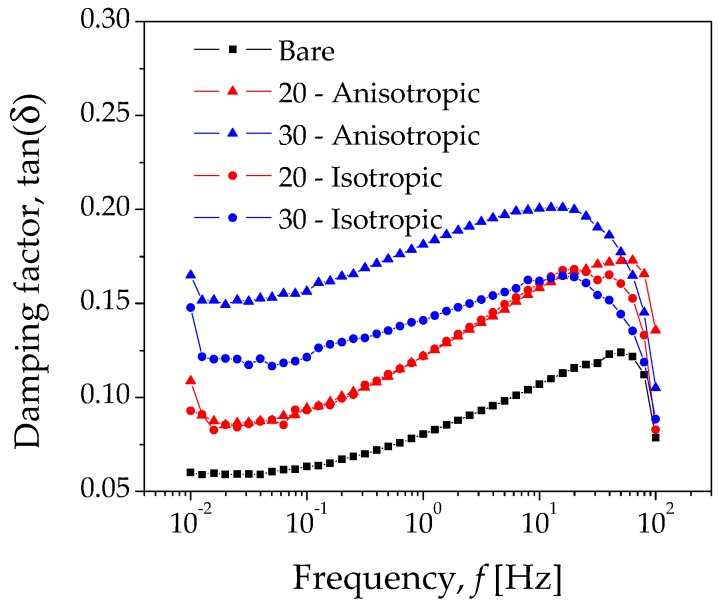
Experimental damping factor for the bare elastomer and the composite MREs.

The experimental curves of $G^{\prime }$ and $G^{\prime \prime }$ shown in Figure 8 are in good agreement with experimental results reported in [31], where a novel shear-stiffened elastomer fabricated with a mixture of silicone rubber and silicon oil were analyzed by dynamic loading conditions, with a parallel-plate rheometer. Furthermore, and from the experimental data illustrated in Figure 8, it is also possible to plot the damping factor dependence, tanδ versus the frequency *f*, as well as the Cole–Cole diagram ($G^{\prime \prime }$ vs. $G^{\prime }$). Figure 9 shows tanδ versus f, which was obtained from the loss-modulus/storage-modulus $\left(G^{\prime \prime }/G^{\prime }\right)$, representing the ratio of loss to the storage moduli during the shear oscillatory experiment. The maximum value observed for the bare sample in Figure 9 corresponds to its peak value observed in Figure 8b.

**Figure 10:**
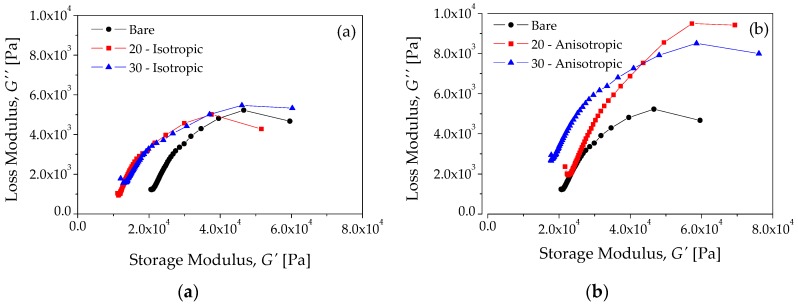
Collected experimental results obtained from: (**a**) the isotropic and (**b**) the anisotropic material samples.

Figure 10 illustrates the Cole–Cole diagrams. In this case, the shape of the experimental curve is similar to the theoretical diagrams plotted in Figure 4b by using Equation (7). This suggests that these experimental results shown in Figure 8, Figure 9 and Figure 10 can be modeled by the FZM. Therefore, the experimental Cole–Cole diagrams can be used to analyze the effect of carbonyl iron microparticles on the rheology behavior of the MREs. Figure 10a shows the Cole–Cole diagrams for the bare sample and for the MRE samples (isotropic dispersion), while Figure 10b shows the Cole–Cole diagrams for the anisotropic material samples.

It can be seen from Figure 10a,b that the mechanical properties improve due to the anisotropic dispersion of carbonyl iron microparticles into the silicone–rubber matrix. In the next section, these experimental results are compared to theoretical predictions obtained from the FZM.

## 5. Comparison between Experimental Results and the Fractional Zener Model’s Theoretical Predictions

**Figure 11:**
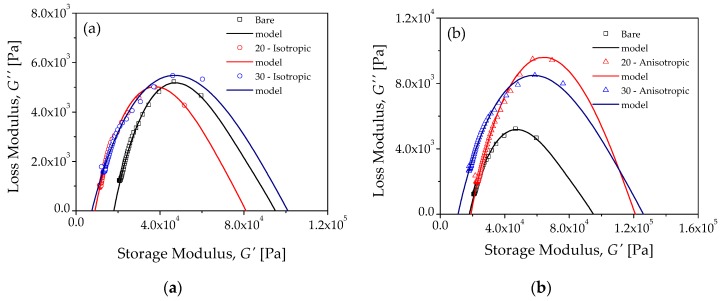
Comparison of experimental data and theoretical simulations of the Cole–Cole diagrams for MRE: (**a**) isotropic samples and (**b**) anisotropic samples.

Figure 11a,b show a comparison between theoretical and experimental results obtained for the MRE-isotropic and MRE-anisotropic samples, respectively. As one can see, theoretical predictions obtained from Equations (6) and (7) follow experimental data well. Table 1 shows the values of fractional orders a and b of the two spring-pots, as well as the parameter values used to obtain the FZM’s theoretical predictions for each material sample, while Table 2 illustrates the amount of error attained between experimental data collected from the composite materials and theoretical predictions. As shown in Table 2, the agreement between experimental results and the theoretical FZM predictions is evident.

The determination of the parameter values *a* and *b* can be done from the Cole–Cole diagrams, as it has been explained in references [22,23,27]. Recall that the fractional parameters a and b represent the molecular mobility associated to the mechanical relaxation process at high and low frequencies, respectively. When values of the fractional parameters are close to 0, the MRE samples have a solid-like behavior, and their molecular mobility is very much localized. On the other hand, when fractional parameters increase (close to 1), the MRE samples display liquid-like behavior, and their molecular mobility is less localized.

Notice that the values of *a* and *b* are such that b>a for all samples studied. This implies that molecular mobility is more localized at high frequencies than for low frequencies. For the bare sample, with no particles added, a= 0.1 and b= 0.275. These fractional values are very close to the fractional parameters calculated for the mean relaxation process of a semi-crystalline polymer at lower temperatures, with respect to its glass transition temperature [23]. The relaxation time parameters associated with the two spring-pots are strongly dependent on temperature [22,23,24]; however, during experimental measurements of $G^{\prime }$ and $G^{\prime \prime }$, the temperature was kept constant, therefore $\tau_{b}$ and $\tau_{a}$ have constant values for all samples studied, with $\tau_{b}$ slightly bigger than $\tau_{a}$.

Since the fractional parameters are associated to molecular mobility during the mechanical relaxation process, the variation of a and b must be associated to interactions between microparticles and the polymer matrix. Table 1 shows that the fractional parameter a (molecular mobility at high frequency) was not affected when the microparticles were isotropically dispersed into the polymer matrix. The same behavior was observed for the two concentrations of 20 and 30 wt % of isotropic MRE samples. However, for anisotropic MRE samples with 20 wt % of carbonyl iron microparticles, the value of a increased from 0.1 (bare sample) to 0.17 (composite sample). For the anisotropic samples with 30 wt % of microparticles, *a* had to be adjusted to the value of a= 0.12. This increment in the value of a corresponds to the MRE-anisotropic samples. This implies that the material molecular mobility at a high frequency is confined. This effect is more pronounced in the molecular mobility at low frequency, because the value of b increases from 0.275 (bare sample) to 0.33, and for the sample with 30 wt % of microparticles, a= 0.285. From the above results, it follows that the value of b is more sensitive than the value of *a*, because the temperature was maintained constantly during experimental measurements of $G^{*}$. In other words, the difference between G′ at low frequency $\left(G_{0}\right)$ and G′ at high frequency $\left(G_{U}\right)$ depends on the effect of the anisotropy and the concentration level of microparticles dispersed into polymer matrix. For the MRE-isotropic sample with 20 wt % of microparticles, $G_{U}-G_{0}$ decreases 5% with respect to the bare sample, and when the concentration level was increased to 30 wt %, $G_{U}-G_{0}$ increased by 23%.

On the other hand, for MRE-anisotropic samples, the increment of $G_{U}-G_{0}$ was more pronounced than for MRE-isotropic samples. For the 20 wt % of MRE-anisotropic microparticles, $G_{U}-G_{0}$ was increased by 34%, and when the concentration level was 30 wt %, the increment obtained for $G_{U}-G_{0}$ was closed to 51%.

The reduction in segmental mobility of the elastomer chains in the composite MRE’s materials is evident in Table 1, in which the values of the parameters *a* and *b* decrease as the microparticle concentration increases. This is an indication of the good adhesion between the elastomer matrix and carbonyl iron microparticles. The damping characteristics of the developed MRE’s composite materials are shown in Figure 9. In this figure, a continuous increase in the experimental values of the damping ratio $\tan\delta =G^{\prime \prime }/G^{\prime }$, as a function of the rheometer frequency for the isotropic and anisotropic material samples, is observed, until each composite material reaches their maximum damping ratio value; then, as the rheometer frequency increases, the damping ratio starts to decrease. This decrease in the damping ratio hinders the mobility of the elastomeric chains, indicating improved interfacial adhesion. This is confirmed by the values of the parameter *a*, listed in Table 1.

## 6. Conclusions

Isotropic and anisotropic MREs reinforced with 20 and 30 wt % of carbonyl iron microparticles were developed to study and model, through the Fractional Zener Model (FZM), their rheological response behavior. It has been found that when the curing process is carried out under the effect of a magnetic field, alignment of microparticles parallel to the magnetic field is obtained. Experimental sample characterization by SEM analysis confirmed the homogeneous particle distribution, as well as their alignment when the material was under the action of a magnetic field.

Collected data from the material samples subjected to angular shear-mode harmonic motion were used to assess the accuracy of Equation (7), to predict the material rheological response behavior. It was found that theoretical isothermal diagrams of $G^{\prime }\left(f\right)$ and $G^{\prime \prime }\left(f\right)$ describe the experimental data well if the fractional parameters *a* and *b* are appropriately adjusted, as shown in Figure 8. Since these parameters a and b are considered as an indirect measure of molecular mobility for the mechanical relaxation phenomena, the Cole–Cole diagrams were plotted to determine their value by fitting theoretical predictions with experimental data. From Figure 8, it is observed that anisotropic materials provide better material rheological performance when compared to the isotropic ones. In fact, the anisotropic material exhibits a higher dissipation energy capacity, and from the design point of view, there are broader storage modulus interval values at which the material can be tuned to improve its performance when compared to the isotropic material [1,2,3].

From the experimental collected data and from the Cole–Cole diagrams, it is concluded that the anisotropic composites material exhibit improvements in the modulus and damping properties due to high level of carbonyl iron microparticles dispersion. In addition, the loss and the storage moduli of the anisotropic MRE’s composite materials increases by about 72% and 23%, respectively, when compared to that of the bare material. This is mainly due to the confinement of part of the elastomer chains between the carbonyl iron microparticles. However, this is not the case for the isotropic MRE’s composite material samples, since there is an evident reduction in the loss and storage moduli values of about 9.4% and 19.4%, respectively, for the composite material with 20 wt % of microparticles, when compared to the maximum recorded values for the bare material. It is also evident that the addition of carbonyl iron microparticles to the bare material enhances the damping properties, and then the energy dissipation capacity of the developed material could be adjusted in accordance with the material application. Therefore, it is concluded that the Fractional Zener Model predicts the isothermal specters of $G^{*}$ well for isotropic and anisotropic MRE’s materials reinforced with carbonyl iron microparticles.

## Figures and Tables

**Table 1:** The Fractional Zener Model (FZM) parameters.

Parameter	Bare	Isotropic Microparticles 20%	Anisotropic Microparticles 20%	Isotropic Microparticles 30%	Anisotropic Microparticles 30%
$G_{0}$ (Pa)	18,000	9000	19,000	7500	11,000
$G_{U}$ (Pa)	94,000	81,000	121,000	101,000	126,000
a	0.1	0.11	0.17	0.1	0.12
b	0.275	0.275	0.33	0.22	0.285
b−a	0.175	0.165	0.16	0.12	0.165
$G_{U}-G_{0}$	76,000	72,000	102,000	93,500	115,000

**Table 2:** Relative error between the values predicted by the FZM and the experimental values.

Material Sample	Particles Weight Percentage [%]	Error [%]
Bare	0	2.9
Isotropic	20	5.4
	30	4.8
Anisotropic	20	3.8
	30	2.8
